# Postpartum hemorrhage - what the interventional radiologist should know

**DOI:** 10.1186/s42155-021-00277-9

**Published:** 2021-12-13

**Authors:** Blaine E. Menon, Claire S. Kaufman, Anne M. Kennedy, Christopher R. Ingraham, Eric J. Monroe

**Affiliations:** 1 Department of Radiology, University of Washington, 1959 Northeast Pacific Street, Seattle, WA 98195 USA; 2 Department of Radiology & Imaging Sciences, University of Utah, 30 North 1900 East, Salt Lake City, Utah 84132-2140 USA; 3 Department of Radiology, University of Wisconsin, 1675 Highland Avenue, Madison, WI 53792 USA

**Keywords:** Postpartum hemorrhage, Interventional radiology, Diagnostic radiology, Uterine atony, Placenta accreta, Embolization, Arteriovenous fistula, Arteriovenous malformation

## Abstract

Postpartum hemorrhage is a leading cause of maternal morbidity and mortality around the world and can be caused by multiple etiologies. Distinguishing between the various etiologies that lead to PPH and identifying high risk features are crucial to implementing effective clinical management. In this review, the diagnostic imaging features and management principles of some of the most important causes of postpartum hemorrhage are discussed, with an emphasis on the pearls and pitfalls when minimally invasive treatment via interventional radiologic techniques are employed.

## Introduction

Postpartum hemorrhage (PPH) can be divided into primary (early) and secondary (late) clinical entities. Early PPH is defined by the American College of Obstetrics and Gynecology as “cumulative blood loss of greater than or equal to 1,000 mL, or blood loss accompanied by signs or symptoms of hypovolemia within 24 hours after the birth process” (Obstet Gynecol, 2017). The definition of late PPH is defined broadly as hemorrhage that occurs from 24 h up to 12 weeks post delivery. (Obstet Gynecol, 2017). Early and late PPH have distinct etiologies. Early PPH most commonly is associated with uterine atony, trauma, placenta accreta spectrum (PAS), or underlying coagulopathy. Late PPH is more often associated with retained products of conception, subinvolution of the placenta, and rarely congenital or post-traumatic high-flow vascular lesions (Obstet Gynecol, 2017; Dossou et al., 2015). Independent of the underlying etiology, several fundamental steps are taken in the initial management of any patient presenting with PPH.

## Initial management

Conservative management of PPH involves both resuscitation for hemorrhagic shock as well as specific therapies guided to achieve hemostasis. These basic steps include monitoring vital signs and determining the appropriate level of care required, establishing adequate intravenous access, resuscitation with fluid and blood products, and providing appropriate analgesia and anesthesia support. The American College of Obstetrics and Gynecology has released massive transfusion recommendations in the setting of PPH to guide the creation of hospital-wide protocols and potential treatments that are available for review (Obstet Gynecol, 2017). Once resuscitation has begun, several conservative therapies are available to achieve immediate hemostasis.

Uterotonics, thrombogenic agents and direct tamponade are all used routinely as conservative management for PPH. Oxytocin is the first line agent, especially if uterine atony is the suspected cause (Evensen et al., 2017). Oxytocin is routinely administered during delivery to reduce the risk of hemorrhage that can occur with placental separation. Increasing the rate of oxytocin infusion stimulates the upper segment of the myometrium to contract thereby constricting the spiral arteries of the gravid uterus (Evensen et al., 2017). During pregnancy physiologic changes occur in the spiral arteries with invasion of the media and endothelium by trophoblasts, dilation of the vessels, and replacement of the normal muscularis layer by a fibrin layer. While this helps with blood flow to the placenta during pregnancy it precludes normal vessel contraction.

**Fig. 1:**
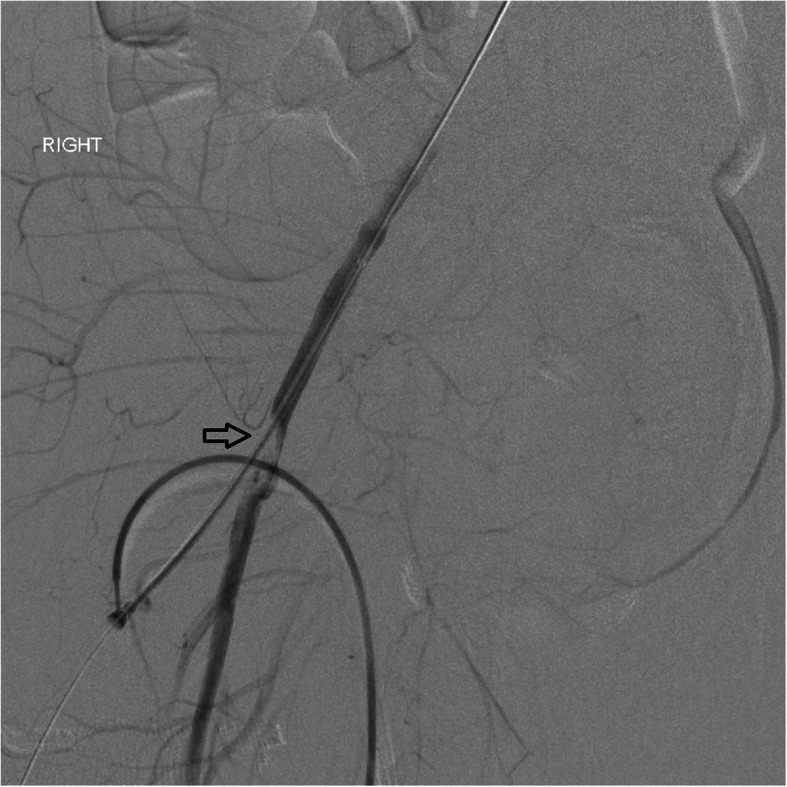
23 year old female with PPH not controlled with Bakhri balloon compression and TXA who presented for uterine artery embolization. Angiogram post embolization showed thrombus in the right common femoral artery at the site of arterial access (arrow)

Direct compression can be employed with the aid of devices like an intrauterine balloon (Bakri balloon) which can function to directly tamponade uterine bleeding (Aibar et al., 2013). Along with uterotonics and compression, a hemostatic agent can be used in the standard treatment of PPH. Tranexamic acid is the agent of choice and is typically given within 3 h of bleeding onset (Evensen et al., 2017). It works by inhibiting the breakdown of fibrin and fibrinogen by plasmin, thus predisposing the patient to a hypercoagulable state. Special care should be taken by the interventionalist, especially in patients who have received transexamic acid, as these patients are prone to thrombosis which can complicate any efforts of treating the underlying cause via percutaneous vascular access (Fig. 1).

If serious postpartum hemorrhage continues after conservative management, escalation of care is warranted. Historically, the recommendations have been to move the patient to the operating suite for surgical control of bleeding, often involving hysterectomy or uterine compression sutures. However, minimally invasive percutaneous treatments offer an alternative strategy to obtain hemostasis and are being offered with increasing frequency. In both of these cases, diagnostic imaging can provide invaluable information regarding etiology, complications, and specific anatomic details for treatment planning. In this review we discuss the high yield imaging features and interventional approaches for managing PPH in general, as well as key points related to three important causes of PPH: uterine atony, placenta accreta spectrum, and vascular injury.

## General approach to PPH

In many cases of PPH the diagnosis can be made with physical exam and history. However, imaging plays a pivotal role in identifying the cause of bleeding when the diagnosis remains unclear after these initial measures. Ultrasound offers many advantages as the initial imaging modality of choice. US is relatively quick, cost-effective, and does not expose the patient to any ionizing radiation.

Computed tomography (CT) with intravenous contrast can also be considered in the evaluation of PPH. Although not recommended as a first line option, CT can serve as a problem solving tool if clinical ambiguity remains after initial work up. In this setting, the strongest recommendation to use CT is in cases of persistent or recurrent bleeding after empiric embolization (Uyeda et al., 2020; Sierra et al., 2012). CTA can determine if active arterial extravasation is present and localize the site of bleeding. Multiphase imaging can also be useful in the detection of vascular anomalies, in addition to providing a more detailed anatomic evaluation of feeding and draining vessels. CT can also be useful in patients with a high suspicion for surgical causes of PPH that would not be amenable to endovascular intervention, such as uterine rupture or vaginal tract laceration (Uyeda et al., 2020). In this regard CT can help triage patients to the appropriate treatment..

MRI is not typically recommended for the evaluation of primary PPH. This modality is limited by its relative lack of access and increased time of image acquisition compared to both US and CT. However in cases of delayed PPH, especially in which the US or CT findings do not offer a clear clinical diagnosis, MRI can be an excellent choice for further work up (Uyeda et al., 2020). MRI has superior soft tissue characterization that increases its sensitivity for identifying a myometrial defect in cases of uterine dehiscence (Leyendecker et al., 2004). Another unique advantage of MRI is the ability to characterize an underlying vascular anomaly without using intravenous contrast through the presence of flow voids with spin echo sequences (Laifer-Narin et al., 2014). This can be useful in specific patients such as those with contrast allergies or renal impairment.

Once the diagnosis is made, the mainstay of treatment is via transcatheter arterial embolization (Ganguli et al., 2011). TAE offers unique advantages over surgical ligation of the internal iliac or uterine arteries because it is able to easily evaluate for and treat extra-uterine sources of the bleed. TAE also spares the patient the high risk of mortality and loss of fertility associated with emergency hysterectomies (Machado, 2011). Another management approach IR can offer is prophylactic balloon catheter occlusion. This can be done in conjunction with TAE and/or surgical hysterectomies, helping maintain hemodynamic stability throughout the procedure to make these procedures safer.

## Approach to uterine atony

Uterine atony is the failure of the uterus to contract effectively after delivery. This is by far the most common cause of early PPH (Reale et al., 2020). As described above, spiral arteries of the gravid uterus lack normally functioning smooth muscles and depend on external compression for hemostasis. Therefore, when the uterus does not contract sufficiently, bleeding from these arteries can lead to early PPH. Risk factors for uterine atony include prolonged or precipitous labor, uterine fibroids, prolonged oxytocin infusion, polyhydramnios, multiple gestations and elevated body mass index (Wetta et al., 2013).

Patient history and physical exam are typically sufficient to make the diagnosis of uterine atony. Clinically these patients have an enlarged, boggy uterus and endorse one or more of the risk factors described above. Physical exam may reveal a well contracted uterine fundus and an atonic lower uterine segment. When imaging is obtained, it is most useful to exclude other causes of PPH as the imaging findings of uterine atony overlap with other causes of hemorrhage. Imaging with CTA can also help confirm whether or not there is active hemorrhage. The accuracy for detecting contrast extravasation via multiphase CT in settings of acute PPH has been reported to be as high as 97% (Lee et al., 2010a). A non-contrast study is important prior to the arterial phase to identify true extravasation especially in cases of vaginal packing. CTA will also demonstrate anatomic variations of the internal iliac, uterine, and ovarian arteries which can guide potential endovascular therapy. In cases of atony however, it is important to consider that the sensitivity may be reduced because of slow-flow or intermittent bleeding, which can lead to false negative exams (Sierra et al., 2012). CT imaging features of uterine atony are also not specific and include contrast extravasation and/or hematoma within the uterine cavity, and an enlarged, heterogeneous postpartum uterus (Lee et al., 2010b).

In cases of uterine atony causing refractory or persistent PPH, the standard of care is to perform a pelvic angiogram followed by bilateral uterine artery embolization. Evidence suggests that uterine artery embolization has a high clinical success rate and is able to achieve hemostasis with obviation of hysterectomy in up to 95% of patients (Chen et al., 2018). Reported complication rates range from 3 to 4.5% (Ganguli et al., 2011; Lee et al., 2012). Minor complications including asymptomatic vascular injury, and hematoma at the puncture site. Major complications that have been reported include endometritis, and pancreatitis, though doubt has been cast on the causal relationship between the procedure and some of these entities given the confounding factor of the ongoing PPH.

**Fig. 2:**
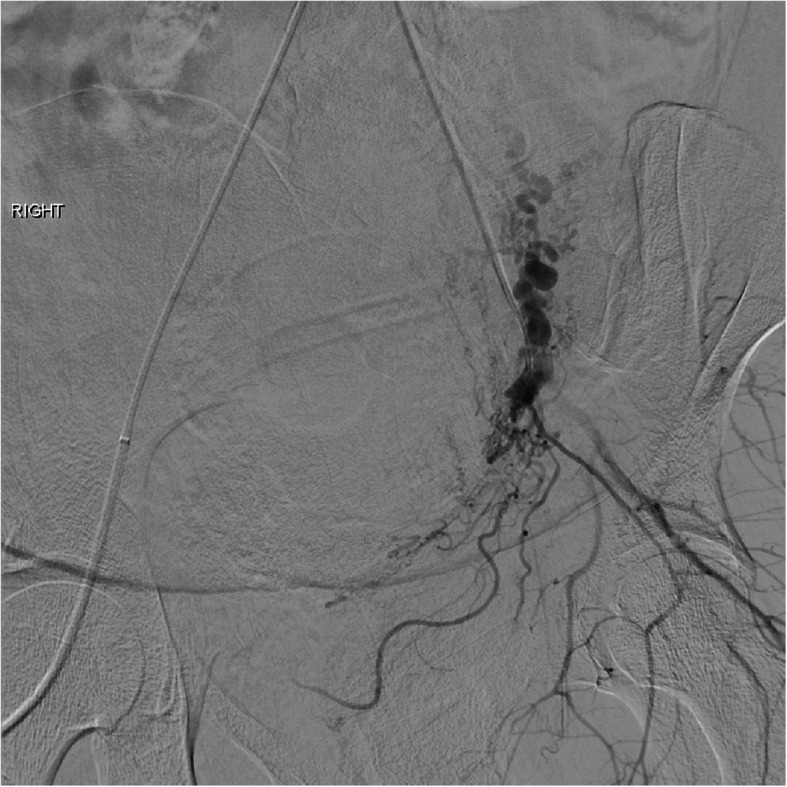
Dilated uterine arteries without evidence of active extravasation in a case of uterine atony

Briefly, our technique for this procedure typically involves a 4- or 5-F vascular sheath placed via the common femoral or radial artery. A flush aortogram may be obtained per the practitioners preferences. Pre-procedure antibiotics are administered if not previously given on labor and delivery. The contralateral internal iliac (hypogastric) artery is selected, localizing the origin of the uterine artery and any potential targets or sites of active extravasation. The angiographic appearance of uterine atony has overlap with that of the normal postpartum appearance, but includes dilated, tortuous uterine arteries, most often without evidence of active extravasation (Fig. 2). The anterior division of the internal iliac is then selected followed by the uterine artery often using a coaxial microcatheter technique. Embolization is typically performed in the distal third or horizontal portion of the uterine artery to avoid vesicular branches. There may be some variety in the embolic agent chosen, however most practitioners prefer a gelfoam slurry given its low cost, high clinical effectiveness and that it acts as a temporary embolic, with recanalization of treated arteries in a matter of weeks. The same steps can be taken to select and embolize the ipsilateral uterine artery.

**Fig. 3:**
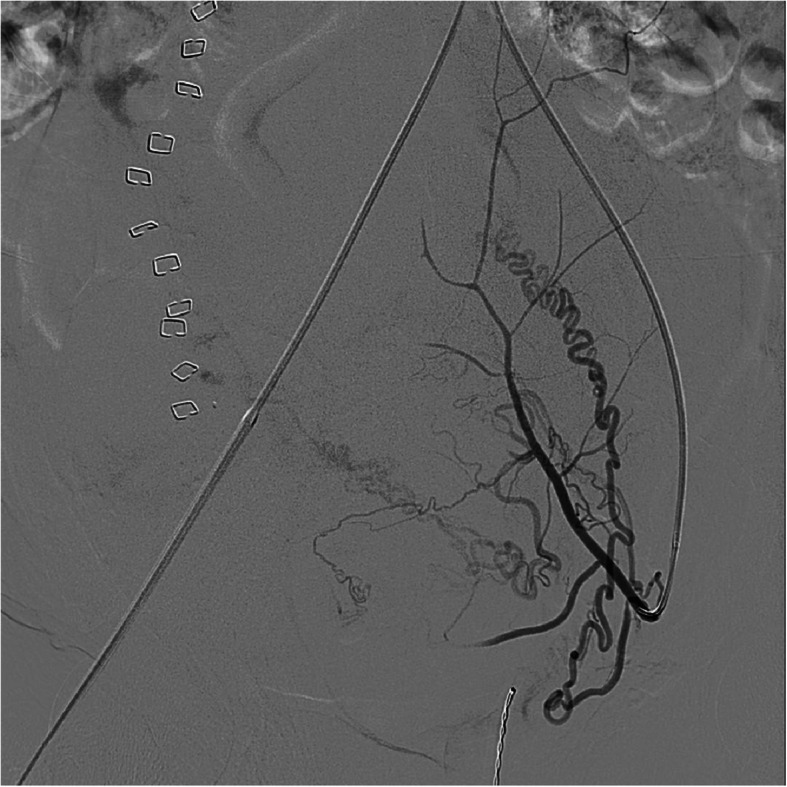
Pelvic angiogram demonstrates prominent round ligament artery (arrows) arising from the inferior epigastric artery and contributing to a case of severe PPH that was subsequently embolized

Collateral arterial pathways hypertrophy during pregnancy and may be investigated empirically or in cases of PPH refractory to hypogastric arterial control. The ovarian arteries arise from the anterolateral aorta at the L2–3 vertebral body level and subsequently descend into the pelvis. Although a well known source of collateral supply to uterine fibroids, they may similarly be responsible for ongoing PPH (Razavi et al., 2002; Wang et al., 2009). The artery of the round ligament (Sampson’s artery), while normally minuscule and angiographically occult, hypertrophies during gestation to provide collateral uterine arterial supply. Their contribution to PPH may be substantial, requiring separate catheterization and embolization (Leleup et al., 2017; Wi et al., 2009). Angiographically, these appear as tortuous arteries originating from the inferior epigastric artery, or less commonly directly from the external iliac artery, and subsequently ascending into the pelvis (Fig. 3).

## Approach to placenta accreta spectrum

Placenta accreta spectrum (PAS) encompasses all situations in which there is abnormal placental attachment. In 2019, the International Federation of Gynecology and Obstetrics proposed an updated nomenclature for PAS with three increasing grades of severity (Jauniaux et al., 2019): Grade 1 - formerly placenta accreta whereby the chorionic villi attach beyond the endometrium without invasion into the deeper myometrium; Grade 2 - formerly placenta increta whereby the villi invade the deeper myometrial layer; Grade 3 - formerly placenta percreta whereby the villi invade fully through the myometrium into the serosa and/or the surrounding structures.

**Fig. 4:**
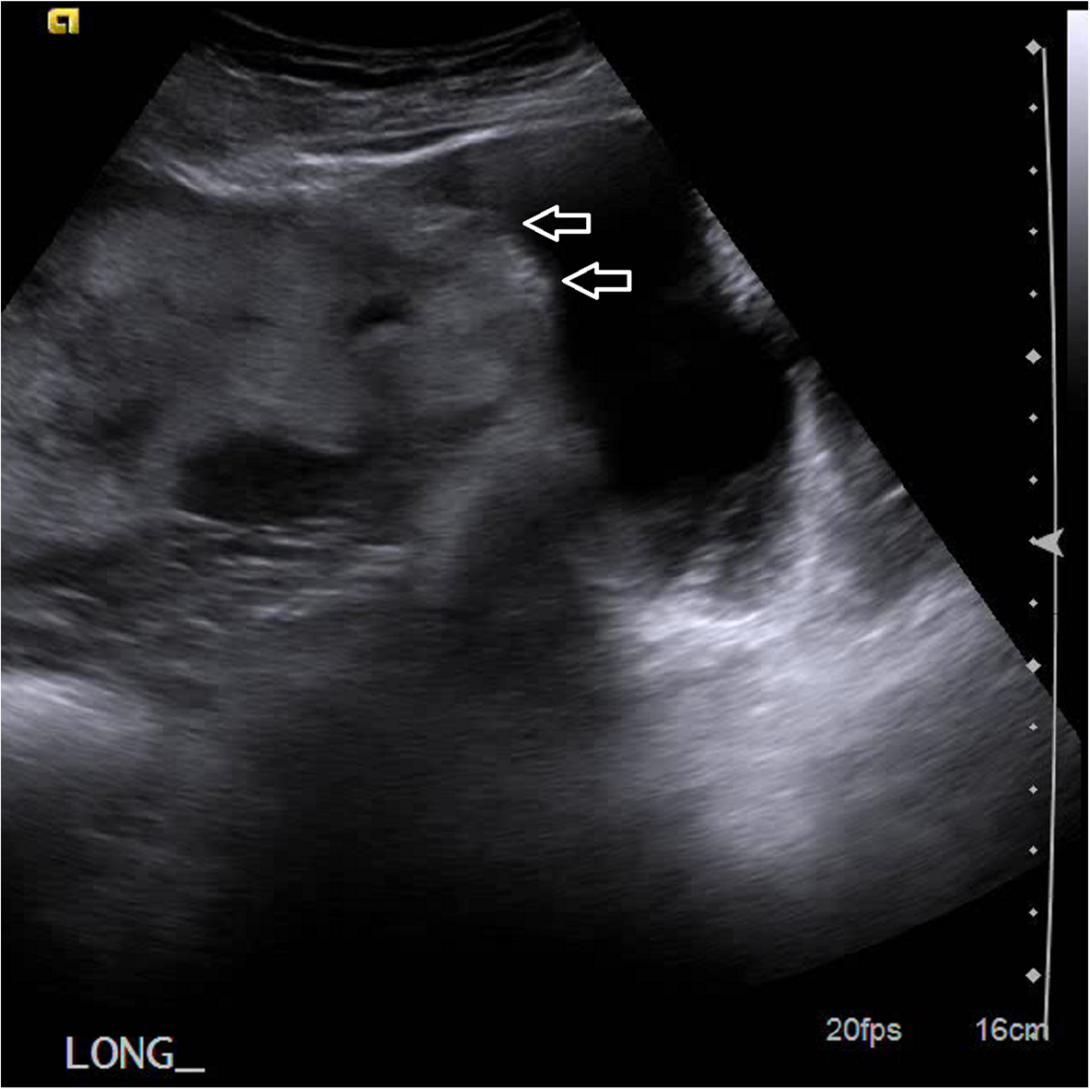
Grey scale sonographic image showing many of the features of PAS, including loss of the retroplacental hypoechoic zone, retroplacental myometrial thickness < 1 mm, and a lower uterine segment echogenic “bulge” (arrows)

Risk factors for PAS include prior cesarean delivery, a history of uterine instrumentation, placenta previa, advanced maternal age, multiparity, uterine anomaly, and history of PAS (Carusi, 2018). Prenatal diagnosis is crucial for appropriate triage of care prior to delivery to a tertiary prenatal center with multidisciplinary teams experienced in treating PAS. US is an excellent modality to make the diagnosis of PAS. There are several characteristic US imaging features that aid in the diagnosis of PAS such as the presence of placenta previa, multiple placental lacunae or “tornado vessels”, loss of the retroplacental hypoechoic zone, retroplacental myometrial thickness < 1 mm, lower uterine segment “bulge”, abnormal bladder interface, and any placental tissue identified beyond the uterine serosa (Cali et al., 2019; Collins et al., 2016) (Fig. 4).

In cases of clinical ambiguity, cross sectional imaging may be employed. MRI in particular has been shown to be useful when US findings are unclear or in cases of a difficult acoustic window, most often from a posterior placenta (Baughman et al., 2008). Some of the most reliable indirect MRI findings of PAS include uterine bulging, heterogeneous placenta, and placental bands. Direct findings include focal interruptions in the hypointense myometrial border at sites of placental invasion (Baughman et al., 2008). The soft tissue resolution of MRI allows for increased sensitivity for extra-uterine invasion in cases of PAS Grade III which can be essential for surgical planning (Palacios Jaraquemada & Bruno, 2005).

Once the diagnosis of PAS is made, the approach to delivery often involves a cesarean hysterectomy between 34 and 36 weeks of gestation. If this diagnosis is not discovered prior to delivery, massive hemorrhage can result at the time of delivery with potential disastrous consequences. Moreover, even when the diagnosis is made appropriately, if the patient desires uterine sparing techniques to preserve future fertility or avoid major surgery, then hysterectomy may not be appropriate. Interventional radiologists can play a pivotal role in the management of these patients with techniques such as uterine artery embolization, internal iliac artery balloon catheter placement and resuscitative endovascular balloon occlusion of the aorta.

**Fig. 5:**
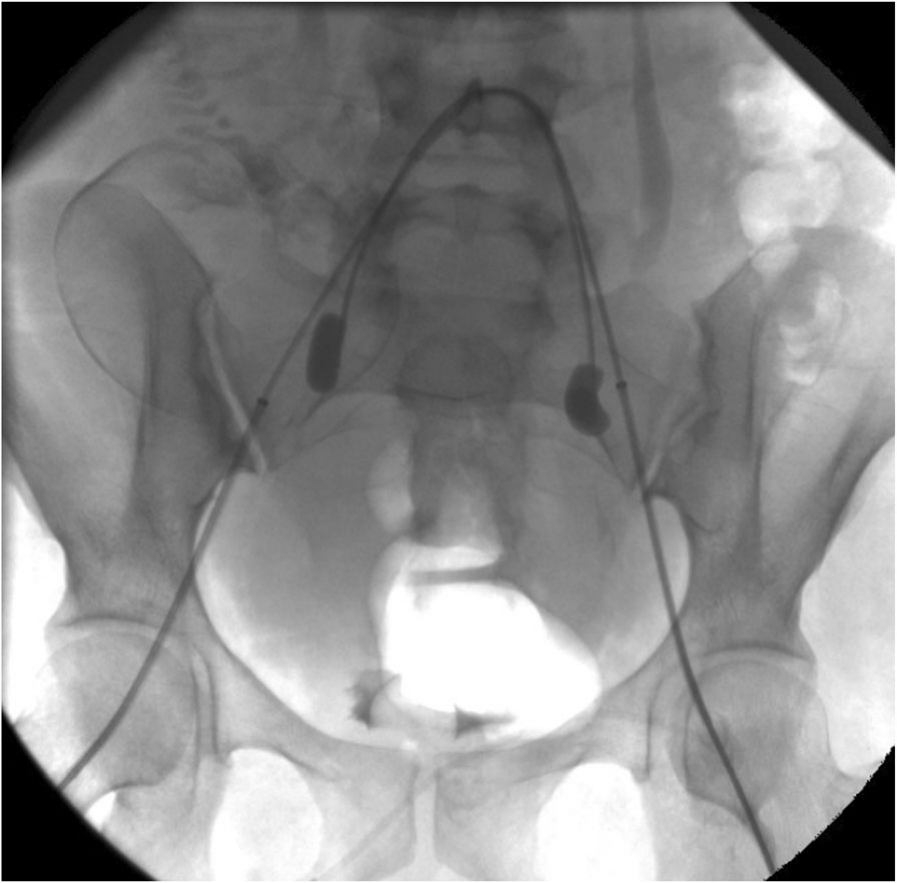
Fluoroscopic spot image demonstrates bilateral common femoral arterial access with 6 french sheath with internal iliac artery balloon placement. Note the fetus is visualized within the pelvis

Internal iliac artery balloon placement is performed prophylactically in planned hysterectomy prior to the cesarean delivery to reduce intraoperative bleeding. The literature surrounding this topic is mixed with conflicting results. Several studies including a systematic review have demonstrated that the procedure is effective in decreasing intra-procedural blood loss, lowering transfusion requirements and decreasing overall morbidity (Nankali et al., 2021). However a randomized control trial from 2020 showed no benefit in morbidity or transfusion requirement (Yu et al., 2020). Our technique involves bilateral groin access via the common femoral artery (Fig. 5). Subselection of the contralateral internal division of the internal iliac artery is made utilizing the operator’s catheter of choice, often a C2. Subsequently, the catheter is exchanged for the balloon occlusion catheter such as a 5 French Fogarty catheter (Edwards Lifesciences Corp, Irving, California). A test inflation of the balloon is performed to confirm position and occlusion. The balloon should be inflated until it elongates slightly and is parallel to the vessel wall with care taken not to oversize or overinflate. The balloon is then deflated and secured in position to the femoral sheath. One advantage of performing the procedure in a hybrid operating suite instead of a separate angiography suite is that the patient does not need to be moved for the two procedures, thereby decreasing the risk of dislodgement of the balloon catheters. The balloons are only inflated during surgery if uncontrolled bleeding is encountered. Inflation can temporarily reduce or stop hemorrhage giving the obstetricians time to obtain control of the bleeding surgically. It is important to note that this technique does not occlude ovarian arteries or other well described collaterals from outside the anterior division of the internal iliac. After hemostasis is achieved the balloons are deflated. In certain high risk patients, it may be useful to leave a single groin sheath in place in case subsequent emergent embolization or angiography is required.

Resuscitative endovascular balloon occlusion of the aorta (REBOA) uses a similar principle, however due to the position of the catheter blood flow through the aorta is occluded, avoiding the issue of extensive collateralization that can happen with internal iliac balloon occlusion. Recent studies have shown this is a promising technique with decreased blood loss, shorter operative times and decreased transfusion requirements (Yu et al., 2020). The technique for this procedure similarly starts with access to a unilateral common femoral artery. The occlusive balloon is placed in the infrarenal aorta using fluoroscopic guidance with test angiography and a predetermined fill volume of the balloon. The balloon is then left deflated in the infrarenal abdominal aorta and secured in position. If uncontrolled hemorrhage is encountered the balloon is inflated to the predetermined size for a set period of time, most often 5–15 min. Given that this technique occludes blood flow to everything distal including the extremities, in order to avoid complications periodic reperfusion must be performed, often in one minute intervals. Advantages to this technique include the shorter procedure time, ease of placement, occlusion of collaterals and the ability to verify appropriate position of the balloon through pulse oximetry of the distal extremity. Overall both of these endovascular techniques are valuable options to improve outcomes in PPH, especially in those cases caused by PAS that ultimately undergo hysterectomy.

## Approach to vascular injuries and anomalies

Another important cause of PPH is the presence of a vascular uterine anomaly, such as those created by iatrogenic trauma. This can be due to inadvertent vessel ligation or blunt trauma and can be seen in either vaginal or cesarean births (Ko et al., 2002). Possible vessels involved include the uterine, ovarian, iliac, and inferior epigastric arteries (Chen et al., 2018). In cases of immediate bleeding, oftentimes this can be directly visualized at the time of delivery. However when the bleeding is refractory or delayed, imaging can serve as an essential tool to find the site of vessel damage.

Vascular injury can be evaluated by Doppler US, which serves as a quick and effective tool to better characterize the type and extent of vascular anomaly. Sometimes, US offers a specific diagnosis. For example in the case of a pseudoaneurysm, just as in other locations, US can reveal an anechoic structure that classically demonstrates the yin-yang appearance of inward and outward flow on color doppler (Chun, 2018). More often, US reveals secondary findings of a vascular anomaly. Typical features on grey scale images include myometrial inhomogeneity as well as tubular spaces within the myometrium. Color Doppler features include multiple enlarged vessels that demonstrate low resistance (RI 0.25–0.55) as well as elevated peak systolic velocities (Timmerman et al., 2003). It is worth noting that some of these features may overlap with multiple causes of shunting including arteriovenous fistula, abnormal placentation, gestational trophoblastic disease, or underlying arteriovenous malformation affecting the uterus. Gestational trophoblastic disease can be easily differentiated from other causes by evaluating serum values of beta human chorionic gonadotropin (beta-HCG). In an arteriovenous fistula or malformation, beta-HCG levels will be low or negative, while in gestational trophoblastic disease beta-HCG levels are expected to be elevated abnormally.

**Fig. 6:**
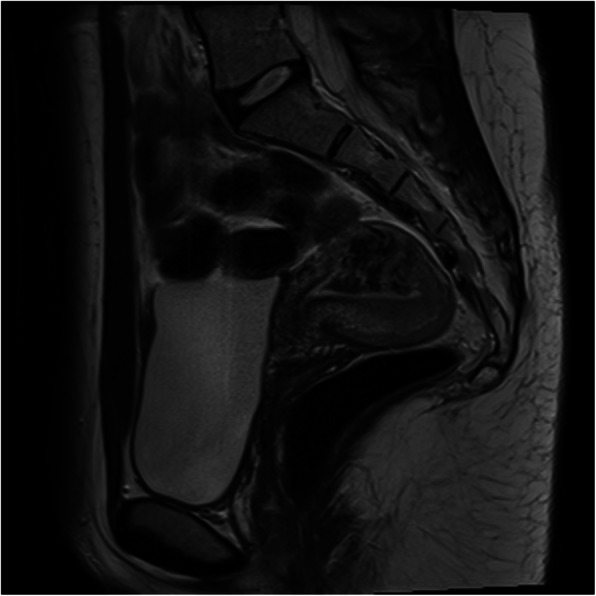
Sagittal T2 weighted image demonstrates a serpiginous cluster of flow voids within the uterine fundus

Cross-sectional imaging either via CTA or MRI/MRA can also be obtained to better define the extent and blood supply of the anomaly and evaluate for extra-uterine involvement. The specific findings depend on the exact type of vascular lesion responsible for the PPH. In cases of arteriovenous fistula, CTA can demonstrate dilated vascular channels and delineate which arteries are providing the abnormal flow (Aiyappan et al., 2014). MRA and time resolved sequences allow for real time evaluation of high flow lesions. Typical findings on MRI include a bulky uterus, disruption of the junctional zones, serpiginous flow related signal voids, and prominent parametrial vessels **(**Fig. 6**)** (Farias & Santi, 2014)**.** This can provide details about blood supply, the vascular nidus, and sites of early venous drainage, all of which are useful for treatment planning.

Treatment choices include hysterectomy, surgical ligation, or endovascular embolization. Endovascular interventions can often be considered first line for reduced patient morbidity, similar to other settings of vascular trauma (Baer-Bositis et al., 2018). Our approach is to access the common femoral or radial artery as done with a prophylactic UAE. On pelvic angiography, any active contrast extravasation, pseudoaneurysm, or abrupt vessel cutoff should qualify as vascular injury related to delivery. If no vascular anomaly is identified at angiography or in cases of hemodynamic instability, we perform empiric embolization of the bilateral uterine arteries with gelfoam.

**Fig. 7:**
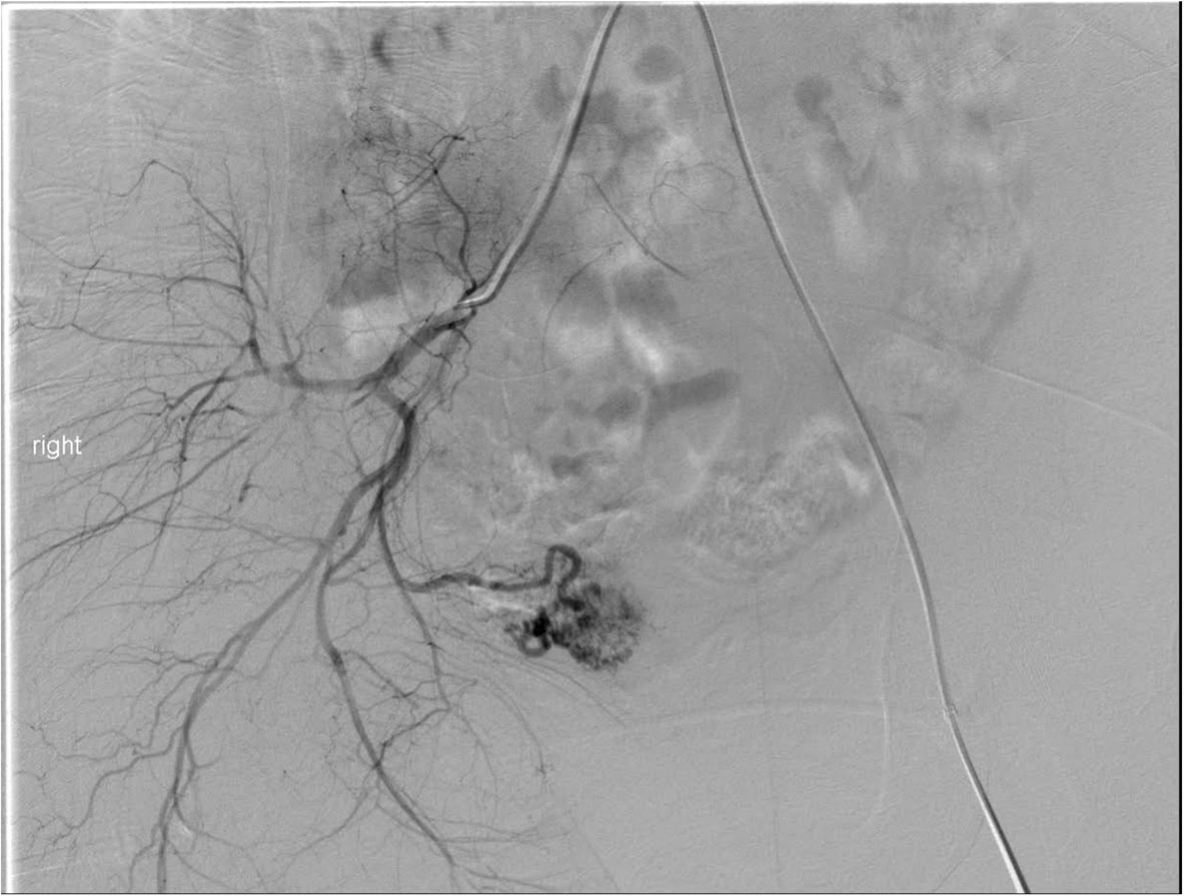
Selective angiography of the right common iliac artery demonstrates an arteriovenous malformation that corresponds to the flow voids seen on the MRI in Fig. 6

**Fig. 8:**
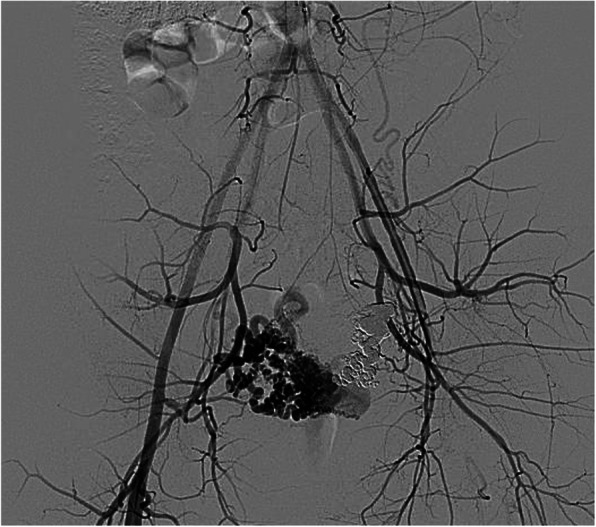
Another case of a known arteriovenous fistula status post Onyx embolization

Uterine AVFs are a relatively rare vascular anomaly that can cause late PPH, often identified on initial imaging as described above. The angiographic appearance of these will often include feeding vessels from bilateral uterine arteries as well as early drainage form the AVF to the pelvic veins **(**Fig. 7**) (Chen et al.,** 2018**)**. Depending on the imaging findings, evaluation of potential extra-uterine feeding arteries may also be pertinent. The choice of embolic is operator dependent, but gelfoam, particles, coils and liquid embolic agents have all been described (Wang et al., 2012; Giurazza et al., 2017; Stiepel et al., 2021; Yoon et al., 2016) (Fig. 8). Our preference is to avoid particle embolics including gelfoam to reduce the theoretical risk of non-target embolization through the shunt. Embolization of uterine AVFs can yield durable long-term results with no evidence of recurrence or residual arteriovenous fistula (Wang et al., 2012; Giurazza et al., 2017; Stiepel et al., 2021; Yoon et al., 2016).

**Fig. 9:**
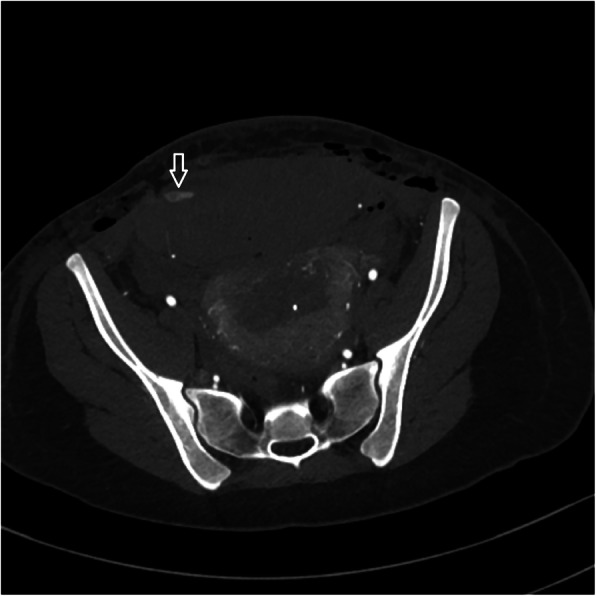
Case of a 16 year old patient status post cesarean section with decreasing hematocrit and hypotension. No signs of vaginal bleeding on exam. CT with active extravasation in the region of the right inferior epigastric artery with large rectus hematoma (arrow)

**Fig. 10:**
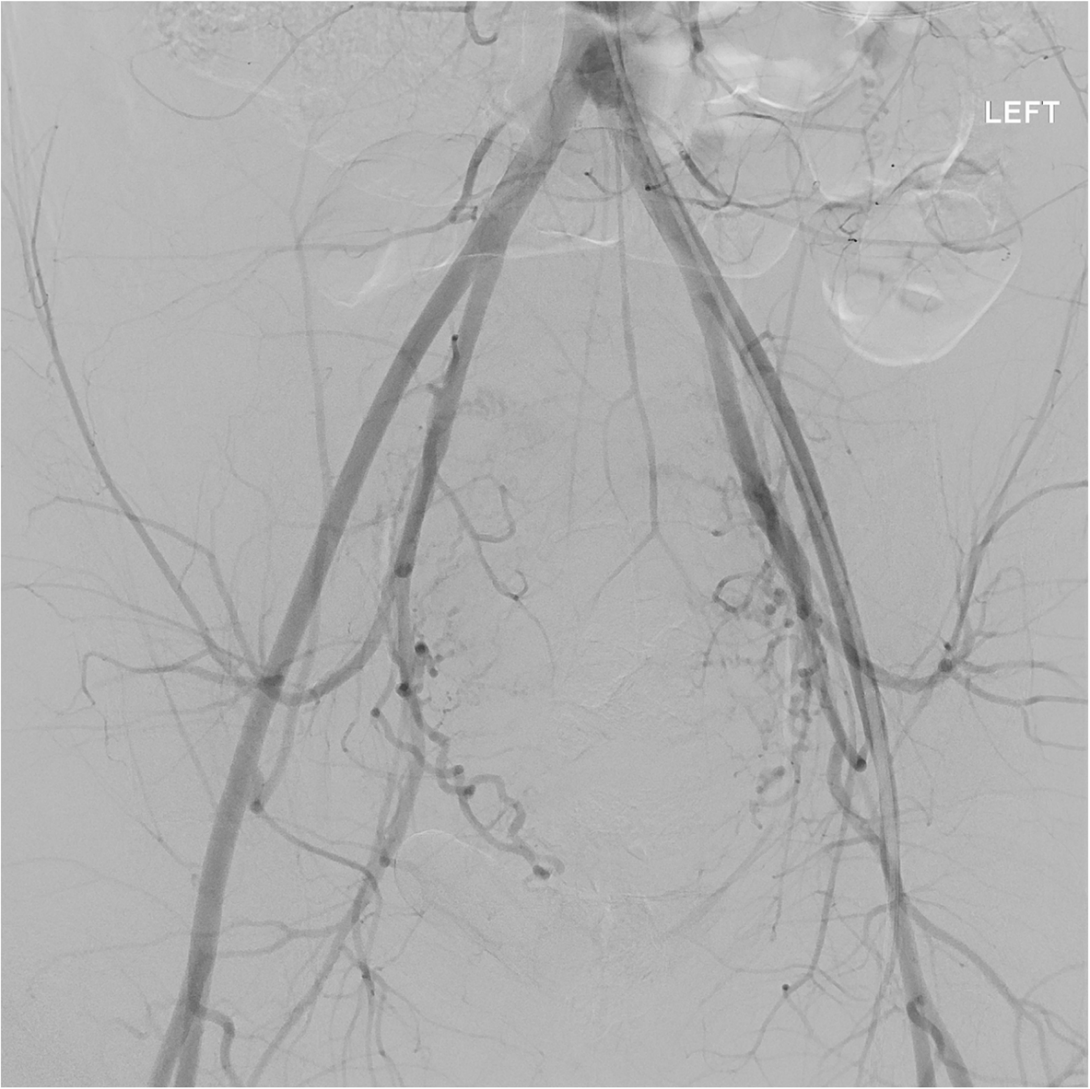
Pelvic angiogram of the same patient as in Fig. 9 demonstrated no active extravasation. Subsequent subselection of the inferior epigastric artery also did not show any active extravasation on angiogram

An additional consideration of vascular trauma unique to cesarean delivery is that of extra-uterine involvement. One particular complication that has been described is the formation of a rectus sheath hematoma (Sufficool et al., 2021) (Fig. 9). These can occur in the setting of injury to the inferior epigastric arteries or one of the smaller perforating branches. Bleeding at this site may be evident based on physical exam of the anterior lower abdomen. However if this is not identified early and the patient is taken to angiography, a site of injury may not be identified on the pelvic angiogram **(**Fig. 10**)**. Prophylactic embolization is often performed even if no active extravasation is seen from the inferior epigastric arteries.

## Conclusions

Postpartum hemorrhage is a serious cause of maternal morbidity and mortality worldwide. The causes and clinical severity of PPH are varied and dependent on a host of complex factors. Diagnostic imaging with US is an important first diagnostic modality to evaluate the various causes and assists with triage and appropriate management. Cross sectional imaging can be useful in complex cases or when clinical ambiguity persists after initial evaluation. For patients in which conservative management fails to achieve hemostasis, minimally invasive percutaneous approaches have become an important treatment option, making the interventionalist an essential member of any comprehensive PPH response team.

## Data Availability

Not applicable.

## Abbreviations

PPH: post partum hemorrhage
PAS: placenta accreta spectrum
CT: Computed tomography
MRI: Magnetic resonance imaging
US: Ultrasound
REBOA: Resuscitative endovascular balloon occlusion of the aorta
UAE: Uterine artery embolization
